# Continued Adaptation of C_4_ Photosynthesis After an Initial Burst of Changes in the Andropogoneae Grasses

**DOI:** 10.1093/sysbio/syz066

**Published:** 2019-10-07

**Authors:** Matheus E Bianconi, Jan Hackel, Maria S Vorontsova, Adriana Alberti, Watchara Arthan, Sean V Burke, Melvin R Duvall, Elizabeth A Kellogg, Sébastien Lavergne, Michael R McKain, Alexandre Meunier, Colin P Osborne, Paweena Traiperm, Pascal-Antoine Christin, Guillaume Besnard

**Affiliations:** 1 Department of Animal and Plant Sciences, University of Sheffield, Western Bank, Sheffield S10 2TN, UK; 2 Laboratoire Evolution & Diversité Biologique (EDB, UMR 5174), CNRS/IRD/Université Toulouse III, 118 route de Narbonne, 31062 Toulouse, France; 3 Comparative Plant and Fungal Biology, Royal Botanic Gardens, Kew, Richmond, Surrey TW9 3AB, UK; 4 CEA - Institut de Biologie Francois-Jacob, Genoscope, 2 Rue Gaston Cremieux 91057 Evry Cedex, France; 5 School of Biological Sciences, University of Reading, Reading RG6 6AH, UK; 6 Department of Biological Sciences, Plant Molecular and Bioinformatics Center, Northern Illinois University, 1425 W. Lincoln Hwy, DeKalb, IL 60115-2861, USA; 7 Donald Danforth Plant Science Center, 975 North Warson Road, St. Louis, MI 63132, USA; 8 Laboratoire d’Ecologie Alpine, CNRS – Université Grenoble Alpes, UMR 5553, Grenoble, France; 9 Department of Biological Sciences, The University of Alabama, 500 Hackberry Lane, Tuscaloosa, AL 35487, USA; 10 Department of Plant Science, Faculty of Science, Mahidol University, King Rama VI Road, Bangkok 10400, Thailand

## Abstract

C$_{4}$ photosynthesis is a complex trait that sustains fast growth and high productivity in tropical and subtropical conditions and evolved repeatedly in flowering plants. One of the major C$_{4}$ lineages is Andropogoneae, a group of $\sim $1200 grass species that includes some of the world’s most important crops and species dominating tropical and some temperate grasslands. Previous efforts to understand C$_{4}$ evolution in the group have compared a few model C$_{4}$ plants to distantly related C$_{3}$ species so that changes directly responsible for the transition to C$_{4}$ could not be distinguished from those that preceded or followed it. In this study, we analyze the genomes of 66 grass species, capturing the earliest diversification within Andropogoneae as well as their C$_{3}$ relatives. Phylogenomics combined with molecular dating and analyses of protein evolution show that many changes linked to the evolution of C$_{4}$ photosynthesis in Andropogoneae happened in the Early Miocene, between 21 and 18 Ma, after the split from its C$_{3}$ sister lineage, and before the diversification of the group. This initial burst of changes was followed by an extended period of modifications to leaf anatomy and biochemistry during the diversification of Andropogoneae, so that a single C$_{4}$ origin gave birth to a diversity of C$_{4}$ phenotypes during 18 million years of speciation events and migration across geographic and ecological spaces. Our comprehensive approach and broad sampling of the diversity in the group reveals that one key transition can lead to a plethora of phenotypes following sustained adaptation of the ancestral state. [Adaptive evolution; complex traits; herbarium genomics; Jansenelleae; leaf anatomy; Poaceae; phylogenomics.]

One of the major goals of evolutionary biology is to understand the origins of key innovations underlying the ecological success of particular groups. This requires the study of adaptive traits in a phylogenetic context, using comparisons of species differing in character states (e.g., Bond et al. 2014; Rainford et al. 2014; McGee et al. 2015; Sánchez-García and Matheny 2017). Because species differ in numerous ways, such comparisons must capture the diversity that emerged after the transition in addition to the diversity that preceded it, to precisely identify the properties directly involved in the origin of the trait of interest (e.g., Sprent 2007; Yukawa et al. 2009; Endress 2011; Puttick et al. 2014; Marek and Moore 2015; Clark et al. 2018). Among flowering plants, C$_{4}$ photosynthesis represents an adaptive novelty with significant ecological consequences (Sage 2004; Edwards et al. 2010; Christin and Osborne 2014).

The C$_{4}$ physiology results from multiple anatomical and biochemical modifications of the ancestral C$_{3}$ photosynthetic metabolism, which include 1) the confinement of the primary enzyme of the photosynthetic carbon reduction pathway, ribulose-1,5-bisphosphate carboxylase/oxygenase (Rubisco), to a compartment isolated from the atmosphere, and 2) increased and cell-specific activity of several enzymes that concentrate CO$_{2}$ at the site of Rubisco (Hatch 1987; von Caemmerer and Furbank 2003; Sage 2004). The concentration of CO$_{2}$ around Rubisco boosts photosynthetic efficiency, and therefore, growth, particularly in high-light, warm and dry conditions (Long 1999; Atkinson et al. 2016).

Although the C$_{4}$ trait requires the modification of multiple components, it has evolved at least 62 times independently during the diversification of flowering plants (Sage et al. 2011). The grass family (Poaceae) encompasses almost half of the C$_{4}$ origins, including some with particular ecological and economic relevance, such as the Andropogoneae (Sage 2017). The roughly 1200 species of this tribe are all C$_{4}$, making it the second-most speciose (Sage et al. 2011) and the most dominant C$_{4}$ lineage (Lehmann et al. 2019). Andropogoneae include some of the world’s most important cereal and fuel crop plants, such as maize, sorghum, sugarcane, and *Miscanthus* spp. (e.g., silver grass), as well as numerous species that dominate tropical savannas and some temperate grasslands, including the tallgrass prairies of the Great Plains (Hartley 1958; Bond et al. 2008; Edwards et al. 2010; Kellogg 2015). Besides generating some of the most productive plants in the world, their C$_{4}$ trait also increased the diversification of Andropogoneae, which in turn has shaped ecosystems around the world (Osborne 2008; Edwards et al. 2010; Forrestel et al. 2014; Spriggs et al. 2014; Sage and Stata 2015). Because 1) they are separated from other C$_{4}$ grass lineages in the phylogeny by several C$_{3}$ branches (Grass Phylogeny Working Group II GPWG II 2012 and 2) the different C$_{4}$ lineages differ in the underlying genetic changes, Andropogoneae are accepted as a C$_{4}$ origin independent from those in other groups of grasses (Sinha and Kellogg 1996; Christin et al. 2008, Christin et al. 2010; Vicentini et al. 2008; Edwards and Smith 2010; Sage et al. 2011; GPWG II 2012; Emms et al. 2016; Gallaher et al. 2019; Niklaus and Kelly 2019).

Due to their economic and ecological importance, Andropogoneae have been included in most studies addressing the evolutionary origins of C$_{4}$ photosynthesis in grasses. In particular, efforts to determine the genomic changes involved in the transition to C$_{4}$ photosynthesis have focused on comparisons between the two C$_{4}$ model Andropogoneae species—maize and sorghum—and distantly related C$_{3}$ model grasses (e.g., rice and *Dichanthelium*; Paterson et al. 2009; Wang et al. 2009; Emms et al. 2016; Studer et al. 2016; Huang et al. 2017). Such a narrow taxon sampling neither covers the diversity of anatomical and biochemical components observed among C$_{3}$ grasses (e.g., Hattersley 1984; Christin et al. 2013; Lundgren et al. 2014) nor among C$_{4}$ grasses within the Andropogoneae themselves (Renvoize 1982a; Ueno 1995; Sinha and Kellogg 1996). Yet, sampling this diversity is crucial for distinguishing those modifications involved in the early origin of the C$_{4}$ pathway in the group as opposed to its subsequent diversification (Christin et al. 2010; Christin and Osborne 2014; Dunning et al. 2017b; Heyduk et al. 2019). Differentiating these scenarios is necessary to determine whether changes for C$_{4}$ evolution were concentrated during the initial history of large C$_{4}$ groups, or whether they were sustained throughout the diversification of large C$_{4}$ clades, as suggested for young C$_{4}$ lineages (Dunning et al. 2017b) and hypothesized based on previously available evidence (Christin and Osborne 2014; Heyduk et al. 2019).

The first divergence within Andropogoneae *sensu*Kellogg (2015) separates the subtribe Arundinellinae from Andropogoneae *s.s.* (tribes Arundinelleae and Andropogoneae, respectively, in Soreng et al. 2017), the latter of which includes the model species maize and sorghum. Until recently, the closest known C$_{3}$ relative of Andropogoneae belonged to a different tribe that also included several C$_{4}$ groups, and the branch separating them was consequently long (Christin et al. 2008; Vicentini et al. 2008; GPWG II 2012; Kellogg 2015; Soreng et al. 2017). However, the C$_{3}$ genera *Jansenella* and *Chandrasekharania* have been recently suggested as the sister group of Andropogoneae based on individual chloroplast or nuclear markers (Besnard et al. 2018; Hackel et al. 2018). High-quality genomes are not available for species from these genera or from Arundinellinae, but low-coverage genome data have recently provided insights into the evolution of the nuclear genome in other nonmodel grasses (Besnard et al. 2014, 2018; Olofsson et al. 2016; Bianconi et al. 2018; Dunning et al. 2019). Capitalizing on the availability of such genomic data sets as a side-product of plastome sequencing (e.g., Washburn et al. 2015; Burke et al. 2016; Arthan et al. 2017; Piot et al. 2018), we are now able to phylogenetically track the modifications underlying one of the major innovations of flowering plants.

In this study, we analyze genome-skimming data for 66 grass species covering the diversity of C$_{3}$ relatives of Andropogoneae, as well as the earliest diversification within the group, to test the hypothesis that C$_{4}$ evolution was sustained throughout the history of old, large C$_{4}$ lineages. First, we generate plastome and genome-wide nuclear phylogenetic trees of grasses to confirm the relationships between Andropogoneae and their C$_{3}$ relatives and estimate the age of C$_{4}$ photosynthesis in the group from a time-calibrated plastome phylogeny. Anatomical traits are then mapped onto the time-calibrated phylogeny to infer the timing of leaf structural transitions in the group. Finally, we look for signatures of adaptive evolution in key C$_{4}$ enzymes, testing whether shifts in selective pressures on protein sequences occurred 1) in a C$_{3}$ context, and therefore, predated the origin of Andropogoneae, 2) at the base of the clade, during a short period of time representing the initial transition from C$_{3}$ to C$_{4}$ photosynthesis, or 3) were sustained throughout the history of the group, representing a prolonged period of gradual innovation within the monophyletic C$_{4}$ Andropogoneae. Overall, our study presents a comprehensive approach to dissecting a complex adaptive trait and inferring the tempo of key phenotypic transitions in a large group of ecological importance.

## Materials and Methods

### Species Sampling and Sequencing

A data set of whole-genome sequences of varied sequencing depth was assembled that covers: the main lineages of Andropogoneae including the subtribe Arundinellinae and the Andropogoneae *s.s.* (*sensu*Kellogg 2015), which represents the earliest known split within this C$_{4}$ group (GPWG II 2012); their putative closest C$_{3}$ relatives; a variety of other C$_{3}$ and C$_{4}$ Panicoideae; and representatives of the other grass subfamilies (Supplementary Table S1 available on Dryad at https://doi.org/10.5061/dryad.j6q573n7x). In total, genomic data for 59 grass species were retrieved from previous studies (Besnard et al. 2013, 2018; Lundgren et al. 2015; Burke et al. 2016; Arthan et al. 2017; Dunning et al. 2017a, 2019; Olofsson et al. 2016; Piot et al. 2018; Silva et al. 2017) and similar data for seven species were generated here (Supplementary Table S1 available on Dryad). For the latter, low-coverage sequencing was performed using Illumina technology. Genomic DNA (gDNA) was isolated from ca. 5–10 mg of leaf material using the BioSprint 15 DNA Plant Kit (Qiagen). Four herbarium samples were sequenced at the Genopole platform (Toulouse, France) while the three remaining samples were extracted from silica-preserved material and sequenced at the Genoscope platform (Evry, France). For all samples analyzed at the Genopole, between 100 and 500 ng of double-stranded DNA were used to construct sequencing libraries with the Illumina TruSeq Nano DNA LT Sample Prep kit (Illumina, San Diego, CA, USA), following the manufacturer’s instructions (for more details, see Besnard et al. 2018). Each sample was multiplexed with samples from the same or different projects and paired-end sequenced on 1/24th of an Illumina HiSeq3000 lane (Supplementary Table S1 available on Dryad). At the Genoscope, libraries were constructed using 250 ng of sonicated gDNA. Fragments were end-repaired and 3’-adenylated. NextFlex DNA barcodes (Bioo Scientific Corporation, Austin, TX, USA) were then added using the NEBNext DNA Modules Products (New England Biolabs, MA, USA) followed by clean up with 1$\times $ Agencourt AMPure XP (Beckman Coulter, Brea, CA, USA). The ligated product was amplified with 12 PCR cycles using Kapa HiFi HotStart NGS library Amplification kit (Kapa Biosystems, Wilmington, MA) followed by a 0.6$\times $ Agencourt AMPure XP purification. Each sample was multiplexed with samples from a different project and paired-end sequenced on 1/48th of an Illumina HiSeq2000 lane (Illumina, USA; Supplementary Table S1 available on Dryad).

### Plastome Analyses

A total of 51 plastome sequences were retrieved from NCBI and another 15 were assembled in this study using the genomic data sets (Supplementary Table S1 available on Dryad). For those assembled here, published plastomes of closely related species (same genus) were retrieved from NCBI and used as references for read mapping using Geneious v.9.1.8 (Kearse et al. 2012; Biomatters Ltd., Auckland, NZ, USA) with the Geneious Read Mapper and default sensitivity. A consensus sequence was then called using the highest-quality base criterion, with indels being manually extended/reduced by the assembly of iteratively mapped reads. In cases where no plastome models were available for congeners, a *de novo* strategy was applied using the software Org.Asm v.1.0 (https://git.metabarcoding.org/org-asm/org-asm) with default parameters. Potential errors in the *de novo* assembly were corrected by mapping the genomic reads to the assembled sequence using Geneious following the strategy described above.

The 66 plastome sequences were aligned with MAFFT v.7.13 (Katoh and Standley 2013), after excluding the second inverted repeat region to avoid representing the same sequence twice (alignments are available on Dryad). Plastome phylogenetic trees were inferred independently for coding and noncoding regions of the alignment using MrBayes v.3.2.6 (Ronquist et al. 2012) with the GTR+G model. Two analyses were run in parallel and were stopped after reaching a standard deviation of splits $<$0.01. A consensus tree was obtained after a burn-in period of 25% and trees were rooted on the BOP clade (Bambusoideae, Oryzoideae, and Pooideae), which is sister to the large PACMAD clade that contains the Andropogoneae (GPWG II 2012). Plastome sequences assembled here were deposited in NCBI (see Supplementary Table S1 available on Dryad for accession numbers).

### Genome-Wide Nuclear Analyses

Because many of the genomic data sets used here have sequencing depths below the minimum required by existing software (Supplementary Tables S1 and S2 available on Dryad; Bertels et al. 2014; Allen et al. 2018), we have adapted pipelines previously used to obtain nuclear phylogenetic trees from genome-skimming data (Olofsson et al. 2016, 2019; Dunning et al. 2017a, 2019). The general approach consists of extracting nucleotide sequences from each genomic data set by mapping reads onto a reference, which is analogous to existing approaches (Allen et al. 2018). However, all positions receiving mapped reads were considered here regardless of the coverage, to allow genome-skimming samples to be incorporated. Sets of coding sequences (CDSs) were used as references, as they represent the portion of the genome that is sufficiently conserved to allow mapping among distant relatives (Olofsson et al. 2016, 2019), and are present at low copy numbers.

A genome-wide reference data set of putative orthologous sequences of grasses was prepared using the complete CDS data sets of three model grasses representing different degrees of divergence from the Andropogoneae: *Sorghum bicolor* (part of the focus group), *Setaria italica* (different tribe in the same subfamily as the focus group), and *Brachypodium distachyon* (different subfamily). These CDSs were retrieved from Phytozome v.12 (Goodstein et al. 2012). Putative one-to-one orthologs were identified using the BLAST reciprocal best hits (RBH) tool as implemented in Galaxy (Cock et al. 2015). Only CDSs that corresponded to the intersection of the RBH among the three species and which were $>$500 bp were retained. Genes potentially transferred from organelles to the nuclear genome were identified via BLAST searches ($e$-value = 10$^{-6})$ using *S. bicolor* organellar genomes as reference and subsequently removed from this data set. The nuclear genome-wide reference data set consisted of 9161 putative orthologs. Each of these genes is expected to descend from a single gene in the common ancestor of the two main groups of grasses, the BOP and PACMAD clades, but might have been lost or duplicated in some derived groups. Collapsing such duplicates allows the extraction of phylogenetically useful markers. Downstream analyses were conducted using the *S. italica* sequence for each group of orthologs, which is closely related, yet outside of the focus group, and therefore, maximizes the evenness of the coverage in the group (Supplementary Fig. S1 available on Dryad).

To minimize the amount of missing data, the nuclear analyses focused on the species of Andropogoneae, Jansenelleae, and Paspaleae for which the estimated sequencing depth was above 1$\times$. *Arundinella nepalensis* was added despite an estimated depth of 0.8$\times $ since it is one of only three Arundinellinae for which sequence data were available. Four species outside of Panicoideae for which high-coverage data were available were added to root the tree. Gene models corresponding to each of the 9161 putative orthologs were assembled independently for each of the 37 grass species included in this reduced data set. First, raw genomic data sets were filtered using the NGSQC Toolkit v.2.3.3 (Patel and Jain 2012) to retain only high-quality reads (i.e., $>$80% of the bases with Phred quality score $>$ 20), and to remove adaptor contamination and reads with ambiguous bases. The retained reads were subsequently trimmed from the 3’ end to remove bases with Phred score $<$20. The cleaned genomic data sets were then mapped as unpaired reads to the genome-wide CDS reference using Bowtie2 v.2.3.2 (Langmead and Salzberg 2012) with default parameters, which map reads identical on 90% or more bases, independently of the read length. Consensus sequences were called based on variant call format files from read alignments with mapping quality score $>$20 using the *mpileup* function of Samtools v.1.5 (Li et al. 2009) implemented in a bash-scripted pipeline, modified from Olofsson et al. (2016, 2019; available on the Dryad data repository). Sites with nucleotide variation among mapped reads were coded as ambiguous bases following IUPAC codes. Consensus sequences shorter than 200 bp were removed from the data set. Sites within each alignment with more than 50% missing data were then trimmed using trimAl v1.4 (Capella-Gutiérrez et al. 2009). Only gene alignments $\ge $300 bp (with $\ge $200 bp per individual sequence) and containing $\ge $50% of the total number of species after trimming were retained for subsequent analyses. Maximum-likelihood (ML) trees were then inferred for each gene alignment using RAxML v.8.2.4 (Stamatakis 2014), with a GTR+CAT substitution model and 100 bootstrap pseudoreplicates. To remove poorly informative markers, gene trees with $<$50% of branches with bootstrap support $\ge $50% were discarded. A multigene coalescent tree using Astral v.5.6.2 (Mirarab and Warnow 2015) was then inferred using the filtered set of gene trees after collapsing branches with bootstrap support values $<$50. Because phylogenomic analyses can be biased by the reference and the amount of missing data (Bertels et al. 2014; Xi et al. 2016; Olofsson et al. 2019), we repeated the mapping and filtering with different filtering stringencies and an alternative reference species (*S. bicolor*; Supplementary Fig. S1 available on Dryad).

In addition to the genome-wide data set, eight individual nuclear markers previously used to infer grass phylogenies (GPWG 2001; Bomblies and Doebley 2005; Doust et al. 2007; Christin et al. 2012b; Estep et al. 2012, 2014) were investigated, namely *aberrant panicle organization 1* (*apo1*), the gene encoding arogenate dehydrogenase (*arodeh*), the DELLA protein-encoding gene *dwarf 8* (*dwarf8*), *floricaula/leafy-like* (*floricaula*), *knotted 1* (*kn1*), the gene encoding phytochrome B (*phyB*), *retarded palea 1* (*rep1*), and the gene encoding granule-bound starch synthase 1 (GBSSI or *waxy*). Sequences of these genes were manually assembled for *Garnotia stricta* var. *longiseta* (Arundinellinae) and the putative C$_{3}$ sister group of Andropogoneae (i.e., *Jansenella* and *Chandrasekharania*) using the reference-guided approach from Besnard et al. (2018). In brief, CDSs from *S. bicolor* were used as seeds to map reads, which were assembled into contigs by recursively incorporating pairs of reads that overlapped with the assembly on at least 30 bp. All sequences of nuclear markers assembled here were deposited in NCBI (see Supplementary Table S3 available on Dryad for accession numbers). Preliminary visualization of read alignments for *Jansenella neglecta* suggested two divergent copies for all genes, but the low sequencing depth for this accession prevented phasing the reads into distinct copies. We, therefore, did not include *J. neglecta* in the phylogenetic analyses of nuclear markers. It is worth mentioning, however, that the short segments that were recovered indicated that one of the copies was very similar to the sequence of *J. griffithiana,* suggesting a hybrid (e.g., allopolyploid) origin of *J. neglecta*. The assembled genomic sequences were aligned with additional data retrieved from NCBI nucleotide databases using MAFFT. Phylogenetic trees were inferred for each of the eight markers using MrBayes, running two parallel chains for 40,000,000 generations. Run convergence and appropriateness of the burn-in period were verified using Tracer v.1.6 (Drummond and Rambaut 2007). The burn-in period was then set to 10,000,000, and a majority-rule consensus was inferred from the posterior trees.

### Molecular Dating

Divergence times were estimated for the plastome data set using a relaxed molecular clock as implemented in BEAST v.1.8.4 (Drummond and Rambaut 2007). The plastome alignment was reduced to CDSs (57,239 bp), to remove intergenic spacers that undergo a large number of insertions and deletions, and are more difficult to align. The phylogenetic tree was time-calibrated by fixing the age of the split between the PACMAD and BOP clades to 51.2 Ma (based on Christin et al. 2014), using a normal distribution with standard deviation of 0.0001. This age represents the scenario based on macrofossils only, but we also report the equivalent ages from a dating scenario including phytoliths (82.4 Ma for the same node; Christin et al. 2014). These microfossils are abundant in the fossil record, but assigning them to modern lineages of grasses is complicated by their restricted number of characters (Prasad et al. 2005, 2011; Christin et al. 2014; Strömberg et al. 2018; for a discussion on the fossil record of grasses, see Kellogg 2015). The GTR+G substitution model was used, with the Yule model as speciation prior and a lognormal uncorrelated relaxed clock (Drummond et al. 2006). Three MCMC chains were run in parallel for at least 250 million generations, sampling every 10,000 generations. The runs were monitored using Tracer v.1.6 (Rambaut et al. 2013) checking for convergence and effective sample sizes $>$100 for all parameters. The burn-in period was set to the point of convergence of the runs (25%) and all trees sampled after that were combined. Median ages were summarized on the maximum clade credibility tree.

### Carbon Isotopes and Leaf Anatomy

Photosynthetic types for most species were retrieved from the literature (Osborne et al. 2014; Supplementary Table S4 available on Dryad). The photosynthetic type of *J. griffithiana* was verified through analysis of carbon isotopes. Leaf fragments from the sequenced herbarium specimen were analyzed using an ANCA GSL preparation module coupled to a Sercon 20–20 stable isotope ratio mass spectrometer (PDZ Europa, Cheshire, UK). The carbon isotopic ratio ($\delta ^{13}$C, in ‰) was reported relative to the standard Pee Dee Belemnite (PDB). Values of $\delta ^{13}$C ranging from $-33$‰ to $-24$‰ are typical of C$_{3}$ plants, and values higher than $-17$‰ indicate that the plants grew using a C$_{4}$ pathway (O’Leary 1988).

Leaf anatomical phenotypes were recorded for members of Andropogoneae and their C$_{3}$ relatives, using data from the literature (Supplementary Table S4 available on Dryad; Renvoize 1982a, Renvoize 1982b, Renvoize 1982c, Renvoize 1985; Watson et al. 1992; Ueno 1995; Zuloaga et al. 2000; Christin et al. 2013). In addition, new leaf cross-sections were prepared for the herbarium samples of *J. griffithiana* and *G. stricta* used for genome sequencing. A leaf fragment (ca. 2 cm) was rehydrated by warming the sample in dH$_{2}$O up to 60 $^{^{\circ}}$C followed by immersion in 1% KOH overnight. The rehydrated fragment was then dehydrated through an ethanol series from 10% to 100% EtOH, with steps of 30 min, and resin-infiltrated with Technovit 7100 (Heraeus Kulzer GmbH, Wehrheim, Germany). Cross-sections of 9 $\mu $m were obtained using a microtome (Leica RM 2245, Leica Biosystems Nussloch GmbH, Nussloch, Germany) and stained with Toluidine Blue O (Sigma-Aldrich, St. Louis, MO, USA). Micrographs were obtained using an Olympus BX51 microscope coupled with an Olympus DP71 camera (Olympus Corporation, Tokyo, Japan). A number of qualitative and quantitative leaf characters related to the C$_{4}$ function were measured on the cross-sections following Christin et al. (2013): number of bundle sheath layers, distance between the centers of consecutive veins (interveinal distance), minimal distance between the bundle sheaths of consecutive veins (bundle sheath distance), fraction of the mesophyll plus bundle sheath area represented by the inner bundle sheath (% inner sheath area), presence/absence of distinctive cells (*sensu*Tateoka 1958; Renvoize 1982b), and localization of starch production.

### Analyses of Protein Sequence Evolution

To test for episodes of adaptive evolution of C$_{4}$ enzymes during different periods of the history of Andropogoneae, branch model tests using the ratio of nonsynonymous to synonymous substitutions rates (dN/dS; Yang 1998; Yang and Nielsen 1998) were conducted on alignments of five genes encoding proteins known to play important roles in the C$_{4}$ pathway (Hatch 1987; Huang et al. 2017): NADP-malate dehydrogenase (NADP-MDH; gene *nadpmdh-1P1*), NADP-malic enzyme (NADP-ME; gene *nadpme-1P4*), phosphoenolpyruvate carboxykinase (PCK; gene *pck-1P1*), phosphoenolpyruvate carboxylase (PEPC; gene *ppc-1P3*), and pyruvate, phosphate dikinase (PPDK; gene *ppdk-1P2*). To test whether shifts in selective pressures could be related to processes other than C$_{4}$ evolution, an alternative set of 12 genes not known to be involved in C$_{4}$ photosynthesis were used as negative controls. These included some paralogs of the same core C$_{4}$ genes for which sequences were available in NCBI database and the individual nuclear markers used for phylogenetic analyses (see above; except *apo1*, *rep1*, and *floricaula*, for which no C$_{3}$ PACMAD species besides *Jansenella* and *Chandrasekharania* was available). For each of these 17 genes, complete or partial CDSs for the putative C$_{3}$ sister group of Andropogoneae and *G. stricta* were manually assembled using the approach described above. Additional sequences were extracted using BLAST (e-value = 10$^{-9})$ from 1) the CDS data set of seven published genomes (*S. bicolor, Zea mays, S. italica*, *Panicum hallii, Panicum virgatum*, *B. distachyon,* and *Oryza sativa*) retrieved from Phytozome 2) the NCBI nucleotide database, and 3) the transcriptomes of 34 PACMAD species retrieved from Washburn et al. (2017). All sequences from each gene were aligned using MAFFT and the alignment was visually inspected. Low-confidence alignment regions containing indels were removed to avoid erroneously inflating estimates of nonsynonymous substitutions. Sequences from multiple accessions of the same species, paralogs, and sequences containing stop codons or frameshift mutations were also removed before the analysis. The third positions of codons were used for phylogenetic inference to decrease biases due to adaptive evolution (Christin et al. 2012a). Phylogenetic trees were obtained using Bayesian inference with MrBayes as described above for the individual nuclear markers. Branch model tests were conducted using the consensus gene trees without collapsing unsupported nodes. These tests were repeated on the species tree obtained from the multigene coalescent analysis (see above), after pruning species for which sequences of the gene were not available. The duplication events inferred from the gene tree were incorporated into this phylogeny by duplicating the corresponding branches. C$_{4}$ species outside Andropogoneae were pruned from all trees before analyses to avoid either 1) inflating the dN/dS estimate for the background branches as a result of independent selection signals in other C$_{4}$ groups, or 2) underestimating dN/dS in the foreground branches by misidentifying the paralog used for C$_{4}$ photosynthesis in these other taxa.

A number of branch models were optimized using *codeml* as part of PAML v. 4.9 (Yang 2007). The null model, which assumes a single dN/dS ratio for all branches, was compared to several branch models that hypothesized a different dN/dS ratio (i.e., shift in the selective pressure) in a set of foreground branches defined *a priori*: 1) the branch leading to Andropogoneae and its C$_{3}$ sister group (shifts in selective pressures before the transition to C$_{4})$; 2) the branch leading to Andropogoneae (shifts in selective pressures during the transition to C$_{4})$; and 3) the branches leading to each of the two main Andropogoneae groups Arundinellinae and Andropogoneae *s.s.* (two independent shifts in selective pressures just after the transition to C$_{4}$). Each model was repeated with a sustained shift in selective pressures from the selected branches to all descendants. The best model was selected using the Akaike Information Criterion, after verifying that it was significantly better than the null model (at a significance level of 5%) as assessed via a likelihood ratio test, with a $P$-value adjusted for multiple testing using the Bonferroni correction.

The number of amino acid substitutions through time was assessed by estimating via ML the branch lengths on the amino acid alignment while constraining the topology to that obtained on third positions of codons. This was performed for all genes analyzed here, using IQ-tree v.1.6.1 (Nguyen et al. 2015) with an automated selection of the model of protein sequence evolution.

## Results

### Plastome and Nuclear Data sets

The plastome alignment of 66 species was 140,427 bp long, of which 56,991 bp corresponded to CDS. The mean estimated sequencing depth for the plastomes ranged from 90 to 4602 reads per site across species. The nuclear data set consisted of 37 species and 365 genes. The alignments were on average 701 bp long (95% range = 370–1532 bp, total = 255,870 bp) and 70% complete (95% range = 63–80%), with an average of 64 parsimony informative sites (95% range = 27–163, total = 23,367; Supplementary Fig. S1 available on Dryad). As expected, less stringent filtering parameters allowed more genes to be retained, and more parsimony informative sites per gene, but resulted in higher amounts of missing data (Supplementary Fig. S1 available on Dryad). The number of genes producing resolved phylogenetic trees was drastically reduced when using the full set of 66 species (Supplementary Fig. S1f–j available on Dryad). The number of genes retained after filtering when using an alternative reference species (*S. bicolor*) was on average 62% higher, but similar patterns of missing data across data sets were observed.

### Plastome and Nuclear Phylogenetic Trees

**Figure 1.**
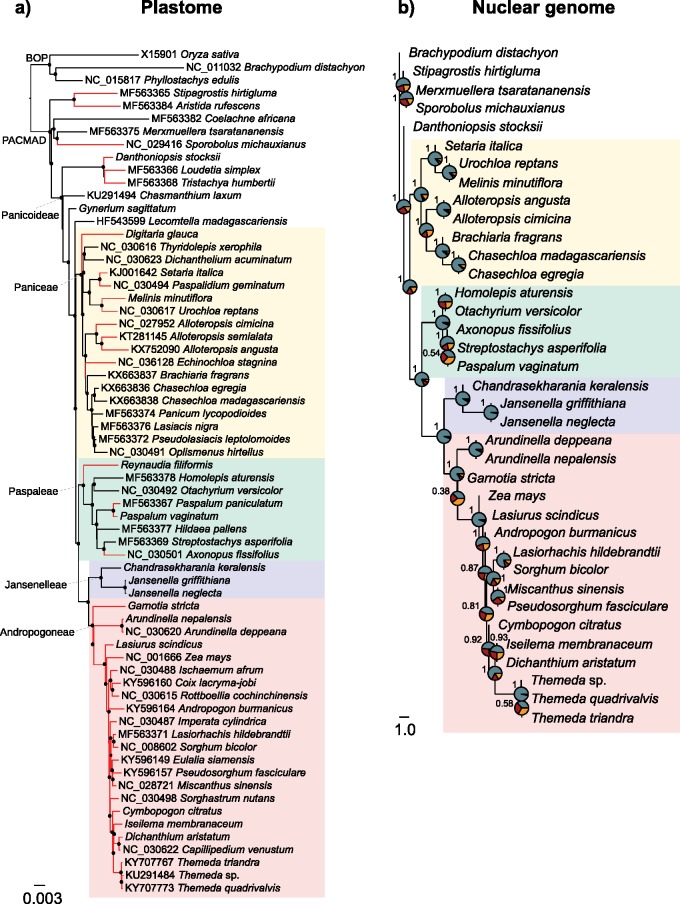
Phylogenetic trees of grasses based on (a) plastomes and (b) genome-wide nuclear data. a) Bayesian phylogram inferred from coding sequences of plastomes (see Supplementary Fig. S2 available on Dryad for phylogram based on non-coding sequences). Branch lengths are in expected substitutions per site. Closed circles on nodes indicate Bayesian posterior probability $\ge.95$. Branches in red lead to C$_{4}$ species. b) Multigene coalescent species tree estimated from 365 nuclear genes. Pie charts on nodes indicate the proportion of quartet trees that support the main topology (in blue), the first alternative (in red), and the second alternative (in orange). Local posterior probabilities are indicated near nodes. Branch lengths are in coalescent units. The major groups of Panicoideae are delimited with shades.

The phylogenetic trees inferred from plastomes and nuclear genomes were largely congruent with previous studies, with discrepancies between the two types of markers as previously reported (GPWG II 2012; Washburn et al. 2017; Moreno-Villena et al. 2018; Dunning et al. 2019). The multigene coalescent tree was generally congruent with the plastid phylogeny. This nuclear analysis revealed gene discordance for many nodes, which indicates incomplete lineage sorting and possibly hybridization in some parts of the family (Dunning et al. 2019). The relationship between Andropogoneae and its C$_{3}$ relatives was however consistent among the plastome and nuclear data sets, with the C$_{3}$ genera *Jansenella* and *Chandrasekharania* forming a strongly supported group sister to Andropogoneae (Fig. 1 and Supplementary Figs S2–S4 available on Dryad). This relationship was also highly supported by all nuclear data sets obtained with different filtering thresholds (Supplementary Table S5 and Fig. S3 available on Dryad), as well as in all trees inferred from individual nuclear markers (Supplementary Fig. S4 available on Dryad), except for two genes, in which *Jansenella* and *Chandrasekharania* formed a paraphyletic group (*apo1*; Supplementary Fig. S4a available on Dryad) or were nested within Andropogoneae (*floricaula*; Supplementary Fig. S4d available on Dryad). Our data and analyses, therefore, provide strong evidence that the clade formed by *Jansenella* and *Chandrasekharania* (hereafter Jansenelleae) is the extant C$_{3}$ lineage most closely related to the Andropogoneae grasses.

Within Andropogoneae, the genera *Garnotia* and *Arundinella* (subtribe Arundinellinae) either formed a group that was sister to Andropogoneae *s.s.* (Supplementary Figs S2–S4f and h and S5b and c available on Dryad), or were paraphyletic (Fig. 1 and Supplementary Figs S4c, d, and g and S5a available on Dryad). Short internal branches, incongruence in the multigene coalescent trees (Fig. 1 and Supplementary Fig. S3 available on Dryad), and low posterior probability support values (Supplementary Fig. S4 available on Dryad) within Andropogoneae $s.s$. are associated with high incongruence between nuclear and plastome trees, suggesting a complex history for the group, which might be related to a rapid radiation and frequent hybridization (Estep et al. 2014). In particular, nuclear and plastome trees identify different taxa as sister to the rest of Andropogoneae $s.s$. (*Zea mays* in the multigene coalescent trees, *Lasiurus scindicus* in the plastome trees; Fig. 1 and Supplementary Figs S2 and S3 available on Dryad).

### Divergence Time Estimates

The confirmation of the sister relationship between Jansenelleae and Andropogoneae allows for refined divergence time estimates, as the most recent divergence from a C$_{3}$ relative (stem group node) represents the upper bound for the origin of a trait that could have evolved at any point along the branch leading to the most recent common ancestor of all species with the trait of interest (crown group node). Divergence times were estimated using the plastome data set (CDSs). Based on a secondary calibration considering only macrofossils, the divergence between Andropogoneae and its C$_{3}$ sister lineage was estimated at 21.1 (95% HPD = 14.6–27.6) Ma (Table 1; Supplementary Fig. S5 available on Dryad). These dates would be pushed back to 34 (95% HPD = 23.5–44.4) Ma if a microfossil dating scenario was followed. The first split within Andropogoneae was estimated at 17.9 (95% HPD = 12.2–23.7) Ma (28.8 Ma under a microfossil dating scenario).

**Table 1.** Divergence time estimates for selected lineages of grasses based on plastome sequences$^{a}$

Clade	Macrofossils$^{b}$	Microfossils$^{c}$
BOP crown	34.7 (24.8–45.6)	55.8 (39.9–73.4)
PACMAD crown	43.4 (34.6–51.1)	69.8 (55.7–82.2)
Panicoideae crown	36.4 (26.3–46.6)	58.6 (42.3–75.0)
Jansenelleae/Andropogoneae split	21.1 (14.6–27.6)	34.0 (23.5–44.4)
Andropogoneae crown	17.9 (12.2–23.7)	28.8 (19.6–38.1)
Andropogoneae $s.s.$ crown	11.9 (8.0–16.0)	19.2 (12.9–25.8)

$^{a}$Median ages are given in million years ago (Ma), with 95% Highest Posterior Density (HPD) intervals in parentheses.

$^{b}$Secondary calibration using Christin et al. (2014) estimates based only on macrofossils.

$^{c}$Secondary calibration using Christin et al. (2014) estimates based on macrofossils plus microfossils.

### Anatomical Changes During the Early Diversification of Andropogoneae

**Figure 2.**
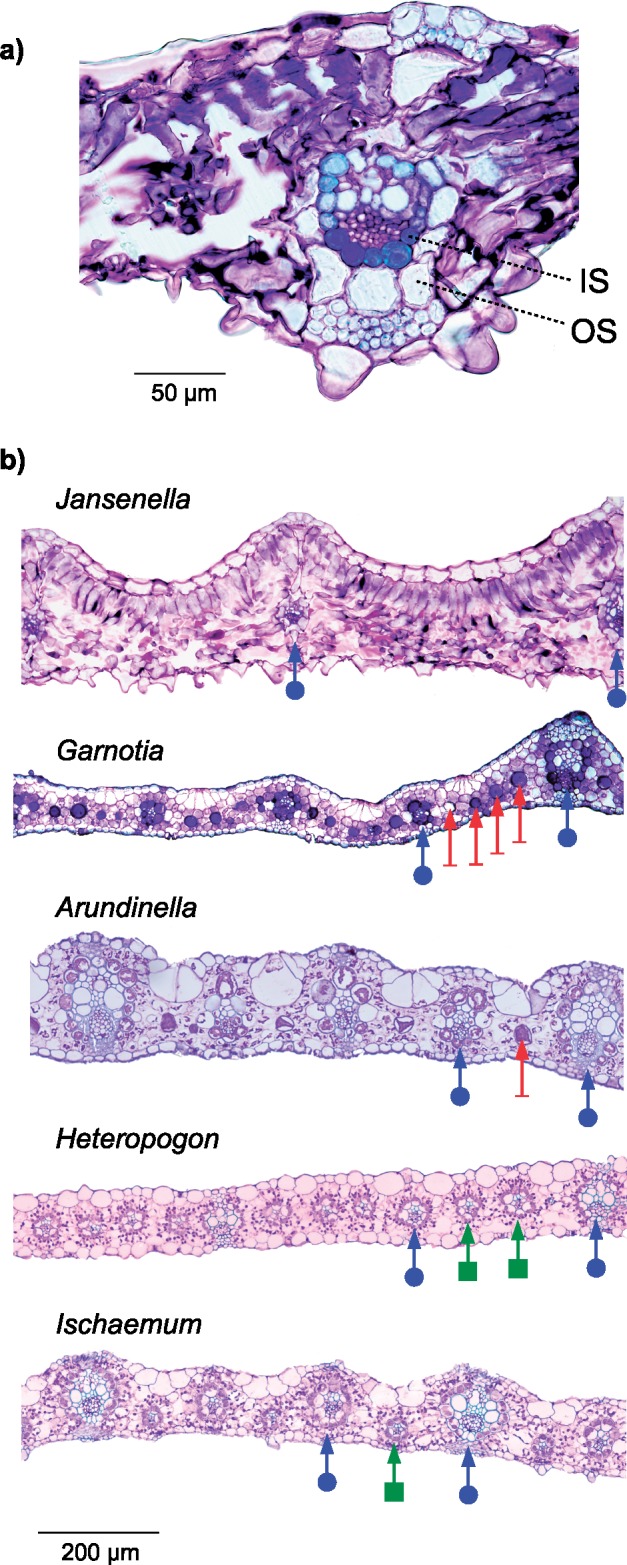
Leaf transverse sections of representatives of Jansenelleae and Andropogoneae. a) *Jansenella griffithiana*. IS, inner bundle sheath; OS, outer bundle sheath. Scale bar = 50 $\mu $m. b) *Jansenella griffithiana*, *Garnotia stricta*, *Arundinella nepalensis*, *Heteropogon contortus,* and *Ischaemum afrum*. The latter three images are from Christin et al. (2013). Arrows with a circle, a dash and a square indicate major veins, distinctive cells, and minor veins, respectively. Scale bar = 200 $\mu $m.

Anatomical and biochemical characters linked to C$_{4}$ photosynthesis were recorded based on the literature and on new measurements for *G. stricta* and *J. griffithiana* (Table 2 and Supplementary Table S4 available on Dryad). Our carbon isotope analysis confirmed that *J. griffithiana* is a C$_{3}$ plant ($\delta ^{13}$C = -27.28%). Its leaf anatomy is typical of C$_{3}$ grasses, with two layers of bundle sheath cells (Fig. 2a), which contradicts previous reports of a single sheath (Metcalfe 1960; Türpe 1970). As with other C$_{3}$ species, it has a large distance between consecutive bundle sheaths, and no minor veins or distinctive cells (Fig. 2b). In addition, the proportion of the leaf occupied by the inner bundle sheath falls within the range observed for other C$_{3}$ grasses (Christin et al. 2013). The leaf anatomy of *G. stricta* was similar to that previously reported for other Arundinellinae (Renvoize 1982b, 1982c; Watson et al. 1992). Its veins are surrounded by a single bundle sheath and are separated by a large number of mesophyll cells (Fig. 2b). Multiple distinctive cells separate the veins, and staining suggests starch production in both bundle sheaths and distinctive cells (Fig. 2b). Similar anatomical structures are observed in other Arundinellinae (Supplementary Table S4 available on Dryad; Renvoize 1982b, 1982c; Watson et al. 1992) but also in the genus *Arthraxon* (Ueno 1995), which is an Andropogoneae $s.s$. representative that diverged early from the rest of the group (e.g., GPWG II 2012; Estep et al. 2014). By contrast, most Andropogoneae $s.s.$ lack distinctive cells and decrease the distance between consecutive veins via the proliferation of minor veins (Fig. 2b; Table 2; Supplementary Table S4 available on Dryad).

**Table 2.** Leaf anatomical traits in *Jansenella griffithiana* and representatives of Andropogoneae

Lineage/species	Bundle sheath layers	Interveinal distance ($\mu $m)	% Inner sheath area	Bundle sheath distance ($\mu $m)	Distinctive cells$^{a}$	Starch in BSC$^{a}$
Jansenelleae (C$_{3})$						
*Jansenella griffithiana*	2	471	0.01	403	A	A
Arundinellinae (C$_{4})$						
*Arundinella nepalensis* $^{b}$	1	215	0.27	101	P	P
*Garnotia stricta*	1	191	0.11	187	P	P
Andropogoneae *s.s.* (C$_{4})$						
*Arthraxon* sp.$^{c,d}$	1	—	—	—	P	P
*Chrysopogon pallidus* $^{b}$	1	112	0.23	29	A	P
*Heteropogon contortus* $^{b}$	1	80	0.21	32	A	P
*Ischaemum afrum* $^{b}$	1	109	0.24	52	A	P
*Sorghum halepense* $^{b}$	1	119	0.20	53	A	P

$^{a}$A = absent, P = present.

$^{b}$ Christin et al. (2013).

$^{c}$ Watson et al. (1992).

$^{d}$ Ueno (1995).

### Positive Selection in C$_{4}$ Enzymes

Phylogenetic trees for genes encoding C$_{4}$ enzymes inferred from third positions of codons were compatible with plastome and nuclear genome trees, with most of the variation being observed within Andropogoneae *s.s.* and Paniceae (Supplementary Fig. S6 available on Dryad). In all cases, Jansenelleae was sister to Andropogoneae, except in *pck-1P1*, where Jansenelleae formed a group with Arundinellinae that was sister to Andropogoneae *s.s.* (Supplementary Fig. S6c available on Dryad). Arundinellinae and Andropogoneae $s.s$. represented the first split within Andropogoneae in *nadpmdh-1P1*, *nadpme-1P4*, and *ppc-1P3*, but not in *ppdk-1P2*, in which Arundinellinae is paraphyletic (Supplementary Fig. S6e available on Dryad) and *pck-1P1,* as mentioned above. Lineage-specific duplications are observed within Andropogoneae $s.s.$ and Arundinellinae species for *nadpme-1P4*, and only in Andropogoneae $s.s$. for *nadpmdh-1P1* (Supplementary Fig. S6a and b available on Dryad), as previously reported (Rondeau et al. 2005; Christin et al. 2009b; Wang et al. 2009).

The inferred trees were used to track shifts in selective pressures in Andropogoneae, independently for each gene. In three core C$_{4}$ genes analyzed (*nadpmdh-1P1*, *nadpme-1P4,* and *ppc-1P3*), the best model inferred an increase of the dN/dS ratio after the split between Andropogoneae and Jansenelleae, which was sustained in the descendant branches (Table 3). As compared to the background branches, the estimated dN/dS ratio was 2–3.7 times higher in Andropogoneae for these genes. In *pck-1P1* and *ppdk-1P2*, the best model indicates two independent three- and four-fold increases of the dN/dS ratio at the base of each of Arundinellinae and Andropogoneae *s.s.*, which was sustained in the descendant branches in *pck-1P1*, but not in *ppdk-1P2*.

**Table 3.** Summary of branch model comparisons

		Branch models$^{a}$							
Scenarios of adaptive evolution			Single episode preceding C$_{4}$ evolution		Single episode during C$_{4}$ evolution, at the base of Andropogoneae		Two episodes during C$_{4}$ evolution, at the base of Arundinellinae and Andropogoneae *s.s.*		
Gene$^{b}$	N$^{c}$	Null model	Basal branch	Sustained	Basal branch	Sustained	Basal branches	Sustained	dN/dS$^{\rm d}$
Core C$_{4}$ genes									
*nadpmdh-1P1* (NADP-MDH)	28	13.53	14.82	5.90	14.74	0.00*	14.55	1.46*	0.07–0.14
*nadpme-1P4* (NADP-ME)	63	205.29	202.70	42.20*	205.26	0.00*	202.73	2.89*	0.08–0.30
*pck-1P1* (PCK)	41	36.77	38.11	5.40*	—	—	38.77	0.00*	0.02–0.06
*ppc-1P3* (PEPC)	51	105.82	107.82	7.83*	82.81*	0.00*	85.87*	21.78*	0.03–0.09
*ppdk-1P2* (PPDK)	30	100.33	97.23	51.07*	83.78*	37.69*	0.00*	53.91*	0.10–0.44
Paralogs of C$_{4}$ genes									
*nadpme-1P1*	30	**1.52**	1.56	0.00	1.28	1.16	3.33	1.73	0.09
*nadpme-1P2*	21	**0.00**	0.72	1.59	2.00	0.97	0.55	0.93	0.09
*nadpme-1P3*	24	10.58	0.00*	4.73	4.77	7.75	9.97	10.05	0.05–0.17
*ppc-1P4*	30	**3.92**	4.08	5.90	5.23	5.30	0.00	3.84	0.07
*ppc-1P5*	30	**0.00**	2.00	1.84	1.83	1.49	1.84	1.80	0.06
*ppc-1P7*	19	11.33	13.29	5.59	—	—	12.65	0.00*	0.09–0.04
*ppdk-1P1*	12	**6.19**	8.17	6.31	7.39	0.37	0.00	0.63	0.18
Other nuclear genes									
*arodeh*	30	**0.00**	1.99	1.70	—	—	1.85	1.77	0.11
*dwarf8*	39	46.51	45.75	23.99*	47.23	2.35*	48.34	0.00*	0.06–0.16
*knotted1*	13	**4.15**	0.00	5.45	—	—	3.60	5.94	0.07
*phyB*	55	**7.95**	4.67	8.35	0.00	9.86	9.71	9.85	0.09
*waxy*	55	**0.00**	1.74	1.70	—	—	1.90	1.91	0.05

*Note*: The best-fit model is highlighted in bold. Asterisks indicate significant likelihood ratio tests (LRT) against the null model after Bonferroni correction. Two hypotheses of potential enzyme adaptation were tested for each scenario, the first assuming a shift in selective pressure only in the basal branch(es) of the group specified (‘Basal branch’), the second assuming a sustained shift from the basal branch including all descendant branches (‘Sustained’). Missing values correspond to trees in which Andropogoneae was not monophyletic.

$^{a}$Delta-AIC values relative to the best-fit model for each gene are shown.

$^{b}$C$_{4}$ gene annotation following Moreno-Villena et al. (2018).

$^{c}$Number of sequences in the alignment.

$^{d}$dN/dS ratios of background and foreground branches, respectively, estimated for the best-fit model, except in cases where the null was the best-fit model, for which there was a single dN/dS estimate for all branches.

A shift of the dN/dS ratio was identified in three of the 12 other genes used as negative controls (Table 3). In the case of *nadpme-1P3*, a gene encoding a NADP-ME isoform not involved in the C$_{4}$ cycle of any of the previously screened species (Moreno-Villena et al. 2018), the best model indicates an increase in dN/dS in the branch leading to the most recent common ancestor of Andropogoneae and Jansenelleae (Table 3). In *dwarf8*, an increase of dN/dS occurred on branches leading to each of the Arundinellinae and Andropogoneae *s.s.* and was sustained in the descendant branches. Finally, the best model for *ppc-1P7*, a gene encoding a PEPC isoform also not co-opted for the C$_{4}$ cycle in any species previously analyzed (Moreno-Villena et al. 2018), assumed a decrease of dN/dS in the two branches leading to each of the Arundinellinae and Andropogoneae *s.s.* and their descendants. Therefore, out of the 12 negative controls, only *dwarf8* presents an increase of dN/dS that coincides with C$_{4}$ evolution in the group. This gene, which affects flowering time (Thornsberry et al. 2001), has been linked to the adaptation of some Andropogoneae to varying climates (Camus-Kulandaivelu et al. 2006).

All positive selection tests were repeated using the multigene coalescent species tree topology (Fig. 1) instead of the trees inferred from third positions of codons (Supplementary Table S6 available on Dryad). The results mostly confirmed those reported above, except that no shift of selective pressure was observed in the core C$_{4}$ genes *nadpmdh-1P1* and *pck-1P1*, nor in *nadpme-1P3* and *dwarf8*. Because sequences of the genes were available for species not included in the species tree, the sampling was reduced in these analyses compared to those based on the gene trees. The reduced evidence for positive shifts might, therefore, reflect a lower statistical power of the tests based on the species tree.

**Figure 3.**
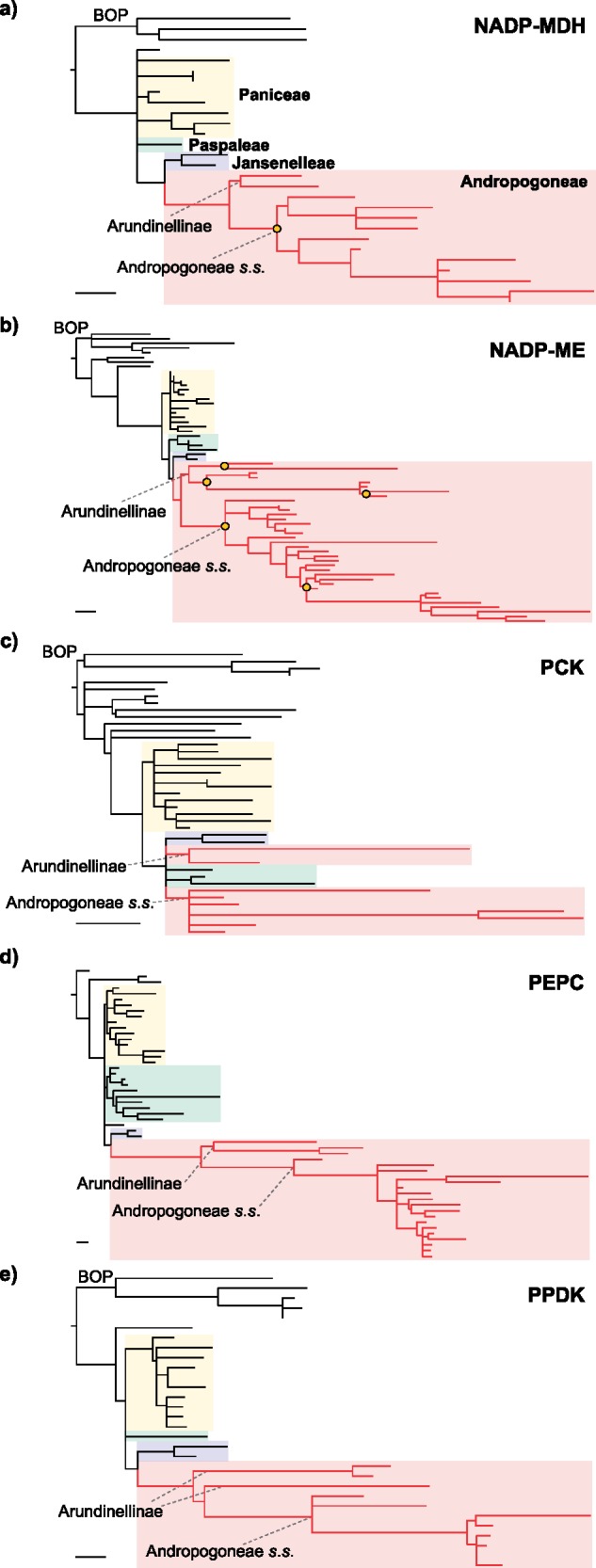
Phylograms with branch lengths based on amino acid sequences. The tree topologies were fixed to those obtained using third positions of codons for genes encoding five core C$_{4}$ enzymes: (a) NADP-malate dehydrogenase (NADP-MDH, gene *nadpmdh-1P1*), (b) NADP-malic enzyme (NADP-ME, gene *nadpme-1P4*), (c) phosphoenolpyruvate carboxykinase (PCK, gene *pck-1P1*), (d) phosphoenolpyruvate carboxylase (PEPC, gene *ppc-1P3*), and (e) pyruvate phosphate dikinase (PPDK, gene *ppdk-1P2*). The major taxonomic groups are indicated with shades, and branches from C$_{4}$Andropogoneae are in red. Yellow circles indicate gene duplications. Scale bars = 0.01 expected amino acid substitutions per site (panels a–e are depicted at different scales). C$_{4}$ species outside Andropogoneae were pruned from the tree. See Supplementary Fig. S7 available on Dryad for details, and Supplementary Fig. S6 available on Dryad for branch lengths based on nucleotides.

**Figure 4:**
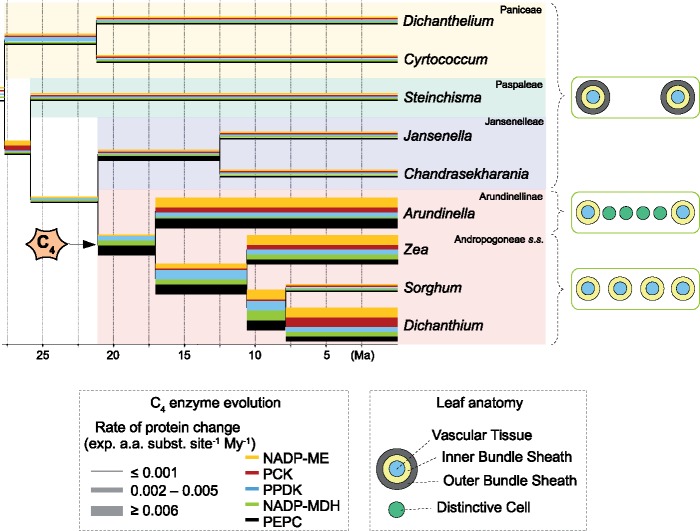
C$_{4}$-related changes in protein sequences and leaf anatomy in the Andropogoneae grasses. A time-calibrated phylogenetic tree of Panicoideae is presented, with selected C$_{3}$ species outside Andropogoneae (see Supplementary Fig. S5 available on Dryad for full phylogenetic tree). Branch thickness is proportional to the rate of protein change and colors represent different C$_{4}$ enzymes. A simplified transverse section of the leaf is presented on the right, with colors representing the different tissues.

To visualize the amount of amino acid substitutions during different periods of the Andropogoneae history, we estimated branch lengths from amino acid sequences after excluding C$_{4}$ species outside of Andropogoneae. Overall, numerous substitutions occurred in *nadpmdh-1P1* and *ppc-1P3* at the base of Andropogoneae, and increased rates compared to non-C$_{4}$ species on these genes were sustained throughout Andropogoneae (Figs 3 and 4). By contrast, bursts of amino acid substitutions in *nadpme-1P4* and *ppdk-1P2* occurred at the base of both Arundinellinae and Andropogoneae $s.s.$ lineages, with the first coinciding with events of gene duplication. An increased number of substitutions is also observed in *pck-1P1*, but it was restricted to a few branches within these groups (Figs 3 and 4). The same patterns were observed when C$_{4}$ species outside Andropogoneae were included in the analyses (Supplementary Fig. S7 available on Dryad), as increased rates of amino acid substitution in all five genes characterize most C$_{4}$ grasses, which highlights the highly convergent nature of C$_{4}$ evolution in grasses. Similar bursts of amino acid substitutions were observed in the negative controls *nadpme-1P3* (on the branch leading to Jansenelleae and Andropogoneae) and *dwarf8* (in a few derived groups within Andropogoneae *s.s.*; Supplementary Fig. S7 available on Dryad).

## Discussion

### A Single Origin of the New C$_{4}$ Physiology Followed by Continued Anatomical Changes

In previous grass phylogenetic trees, Andropogoneae formed a large clade entirely composed of C$_{4}$ species, and its closest known C$_{3}$ relatives belonged to a different group containing multiple independent C$_{4}$ lineages (GPWG II 2012; Gallaher et al. 2019). The branch leading to Andropogoneae was, therefore, long, preventing the precise inference of changes leading to C$_{4}$ evolution in this group. We confirm here that *Jansenella* and *Chandrasekharania* form the sister group of Andropogoneae, both based on plastomes and on markers spread across the nuclear genomes (Fig. 1 and Supplementary Figs S2–S5 available on Dryad). This, combined with a distinctive morphology, supports their recognition as a separate tribe, Jansenelleae (Appendix 1). We further confirm that the group is C$_{3}$, as previously suggested (Türpe 1970; Renvoize 1982a, 1985), providing a shorter branch connecting the last known C$_{3}$ ancestor of Andropogoneae (most recent ancestor shared with Jansenelleae) and the first split within the group. The anatomy of *Jansenella* is typical of C$_{3}$ grasses, with a large distance between consecutive veins, a double bundle sheath, and no minor veins or distinctive cells (Fig. 2). In addition, the genes encoding C$_{4}$-related enzymes from *Jansenella* and *Chandrasekharania* are similar to those of other C$_{3}$ grasses, with no trace of positive selection or increased rates of amino acid replacement (Figs 3 and 4 and Supplementary Fig. S7 available on Dryad; Table 3). We, therefore, conclude that the last common ancestor of Jansenelleae and Andropogoneae was a typical C$_{3}$ plant, with the anatomical and genetic characteristics common to all PACMAD grasses (Christin et al. 2013; Emms et al. 2016; Moreno-Villena et al. 2018). The changes responsible for the emergence of a C$_{4}$ pathway, therefore, happened after the divergence between Andropogoneae and Jansenelleae. Previous studies comparing C$_{3}$ and C$_{4}$ anatomical traits or genomes typically sampled only a few Andropogoneae species, preventing them from assigning changes to different phases of C$_{4}$ evolution (Christin et al. 2013; Emms et al. 2016; Huang et al. 2017), as enabled here thanks to our denser species sampling.

The comparison of anatomical types suggests multiple modifications during the early diversification of Andropogoneae. All species in this group have a single bundle sheath (Renvoize 1982a), which is ontogenetically equivalent to the inner sheath of C$_{3}$ grasses (i.e., the mestome sheath; Dengler et al. 1985). The large distance between consecutive veins, as observed in *Jansenella* (Table 2), is reduced in Arundinellinae by the insertion of one or multiple distinctive cells, where Rubisco can be segregated (Fig. 2; Dengler and Dengler 1990; Sinha and Kellogg 1996). While these distinctive cells are shared by some Andropogoneae $s.s.$ (Ueno 1995), most use a different strategy to reduce the distance between consecutive veins, which consists of the proliferation of minor veins (Table 2; Lundgren et al. 2014, 2019). Distinctive cells and minor veins have similar developmental patterns (Dengler et al. 1996), and the former could be precursors of the latter, in which case minor veins could represent the specialization of ancestral distinctive cells after the split of Andropogoneae $s.s.$ from Arundinellinae. Alternatively, the ancestral state of the group could be minor veins that later degenerated in Arundinellinae and some Andropogoneae *s.s.*, or else these specializations evolved multiple times during the early diversification of the group. In all cases, the phylogenetic distribution of distinctive cells and minor veins shows that changes following the initial transition to C$_{4}$ led to diverse anatomical solutions for the effective segregation of biochemical reactions.

### Modifications of C$_{4}$ Enzymes Occurred Throughout the Diversification of Andropogoneae

The emergence of a C$_{4}$ pathway generally requires the co-option of multiple enzymes already existing in the C$_{3}$ ancestor via their massive upregulation (Hibberd and Covshoff 2010; Moreno-Villena et al. 2018). This is followed by adaptation of their kinetics for the new catalytic context through numerous amino acid replacements (Bläsing et al. 2002; Tausta et al. 2002; Christin et al. 2007; Huang et al. 2017). Tests of shifts in selective pressures conducted here for multiple C$_{4}$-encoding genes from Andropogoneae and other grasses confirm that the evolution of C$_{4}$ genes in this group involved an increased fixation of nonsynonymous mutations (Table 3; Christin et al. 2007, 2009b; Wang et al. 2009; Huang et al. 2017). Genes for the key enzyme of the C$_{4}$ pathway, PEPC, underwent convergent changes in numerous groups of grasses, and most were shared between *Arundinella* and Andropogoneae $s.s.$ (Christin et al. 2007). However, only a fraction of the changes is also observed in *Garnotia stricta* (data not shown), indicating that the enzyme underwent adaptive changes both before and after the diversification of major Andropogoneae lineages. Consistent with this, the branch models did not favor increased amino acid replacements solely at the base of the whole clade, but a sustained increase throughout the diversification of the group (Table 3). A sustained shift in selective pressure on the branch leading to Andropogoneae after the split from Jansenelleae is also supported for genes encoding NADP-MDH and NADP-ME, while independent shifts are observed at the base of Arundinellinae and Andropogoneae *s.s.* for PCK (Table 3). A comparison of branch lengths indeed shows increased rates of amino acid replacements at the respective branches for all five core C$_{4}$ genes (Fig. 3). Our analyses, therefore, confirm that massive changes happened at the base of Andropogoneae, and models assuming that increased fixation of nonsynonymous mutations persisted after early shifts in selective pressures are strongly favored for four out of the five core C$_{4}$ genes analyzed (Table 3). In addition, increased rates of sustained amino acid replacements are observed on many branches within the group (Figs 3 and 4). Increased rates of amino acid replacements were also detected in genes not known to be directly involved in C$_{4}$ biochemistry, such as the NADP-ME paralog *nadpme-1P3*, and *dwarf8*. While the selective drivers for changes in *nadpme-1P3* are not known, the branches with elevated rates of nonsynonymous mutations do not strictly coincide with the C$_{4}$ phenotype (Supplementary Fig. S7 available on Dryad; Table 3). The gene *dwarf8* is linked to flowering time in maize, and selective sweeps in the genomic region including *dwarf8* have been associated with climatic adaptations in maize (Camus-Kulandaivelu et al. 2006, 2008). We conclude that, while other genes undergo elevated rates of amino acid substitutions for different reasons, important alterations of enzymes for the initial build-up of a C$_{4}$ cycle at the base of Andropogoneae were followed by continued adaptation throughout the diversification of the group.

While some enzymes participate in all biochemical variants of the C$_{4}$ cycle (Kanai and Edwards 1999), the identity of the enzyme(s) responsible for the decarboxylation of CO$_{2}$ in the bundle sheath varies among C$_{4}$ lineages (Prendergast et al. 1987; Sage et al. 2011). Our analyses concordantly indicate that the decarboxylating enzyme PCK underwent rounds of amino acid replacements only in some derived groups within Andropogoneae (Figs 3 and 4), without evidence of positive selection at the base of the whole group (Table 3). This conclusion was reached previously (Christin et al. 2009a) and supports later additions of a PCK-catalyzed decarboxylation reaction in some of the Andropogoneae (Gutierrez et al. 1974; Walker et al. 1997; Wingler et al. 1999). However, our data also indicate that NADP-ME, which is the main decarboxylating enzyme in all Andropogoneae, similarly acquired its C$_{4}$ properties relatively late in the history of the group. Again, the best model assumed adaptive evolution throughout Andropogoneae (Table 3). The gene *nadpme-1P4* for NADP-ME was duplicated independently in Andropogoneae $s.s.$, *Garnotia* and *Arundinella*, and amino acid replacements are especially prevalent in one of the copies in each group (Fig. 3 and Supplementary Fig. S7 available on Dryad; Christin et al. 2009b). These observations point to independent adaptation of the enzyme kinetics, but the expression patterns also likely evolved independently in Andropogoneae $s.s.$ and Arundinellinae. Indeed, modifications of the promoter regions allowing the C$_{4}$-specific binding of a transcription factor are restricted to one of the Andropogoneae $s.s.$ duplicates that fulfills the C$_{4}$ function (Borba et al. 2018), which evolved after the split from Arundinellinae. We, therefore, hypothesize that the common ancestor of the Andropogoneae performed a C$_{4}$ cycle based on several decarboxylating enzymes relatively abundant in many C$_{3}$ grasses (Moreno-Villena et al. 2018), with some amino acid changes in the other C$_{4}$ enzymes. Further modifications, which canalized the use of the NADP-ME encoded by *nadpme-1P4*, added a PCK shuttle and/or improved the action of PEPC, PPDK and NADP-MDH happened later during the diversification of the group, so that its numerous C$_{4}$ species represent a diversity of realizations of the C$_{4}$ pathway. Similar conclusions were reached for small groups that evolved the C$_{4}$ trait more recently (Dunning et al. 2017b), but we show here for the first time that the continuous adaptation of the C$_{4}$ trait can be sustained over long evolutionary periods, leaving traces even within one of the largest C$_{4}$ groups.

### $C_{4}$ Physiology Evolved During the Early Miocene in Andropogoneae

Besides inferring the changes underlying C$_{4}$ evolution in Andropogoneae, our plastome phylogeny encompassing a diversity of Andropogoneae and their closest C$_{3}$ relatives shed new light on the age of C$_{4}$ photosynthesis in the group. Our molecular dating estimated the split between Jansenelleae and Andropogoneae at roughly 21 Ma, with the first split within Andropogoneae at 18 Ma. While older ages would be inferred if disputed microfossils dates are considered (see Results), these dates represent the interval in which C$_{4}$ most likely evolved in this group, and are consistent with those obtained from previous studies (Christin et al. 2008; Christin and Osborne 2014; Vicentini et al. 2008; Estep et al. 2014; Spriggs et al. 2014; Dunning et al. 2017a).

Reconstructing the ancient biogeography of Andropogoneae is complicated by their diversity and presumably numerous long-distance dispersals, but India represents the center of diversity of both Andropogoneae and Jansenelleae (Bor 1955; Hartley 1958; Nair et al. 1982; Yadav et al. 2010), suggesting an origin on the subcontinent. The three species of Jansenelleae occur in open habitats (Bor 1955; Nair et al. 1982; Yadav et al. 2010), including some that regularly burn (Shilla and Tiwari 2015), calling for more research to establish which ecological traits now typical for Andropogoneae had already emerged before the C$_{3}$ to C$_{4}$ transition and which only appeared afterwards.

The contrast between the sister groups Jansenelleae and Andropogoneae is striking. While the former has only three known species, two of them restricted to small regions of India, the latter encompasses roughly 1200 species spread around the world, many of which are dominant in savanna ecosystems (Hulbert 1988; Solbrig 1996; Bond et al. 2003; Kellogg 2015). This difference is partially explained by the divergence of photosynthetic types, but the expansion of C$_{4}$ grasslands happened 10–18 Myr after C$_{4}$ originated in Andropogoneae (Edwards et al. 2010), and increased diversification occurred only in some of its subclades (Spriggs et al. 2014). While the initial C$_{4}$ trait might have played the role of a key innovation broadening the niche of early Andropogoneae (Lundgren et al. 2015; Aagesen et al. 2016), the later diversification and dominance of some subgroups, their rapid dispersal across large distances (Dunning et al. 2017a) and into different ecosystems (Watcharamongkol et al. 2018) were likely enabled by the acquisition of additional attributes. Traits only partially related or entirely unrelated to C$_{4}$ photosynthesis, such as frequent allopolyploidy, herbivore resistance, and fire tolerance have previously been used to explain the success of some Andropogoneae (Stebbins 1975; Bond et al. 2003; Edwards et al. 2010; Visser et al. 2012; Estep et al. 2014; Forrestel et al. 2014; Ripley et al. 2015; Linder et al. 2018). We suggest that the diversity of C$_{4}$ phenotypes revealed here might also contribute to variation among Andropogoneae. For instance, the addition of a PCK shuttle, which happened recurrently in some derived Andropogoneae, is predicted to increase tolerance to fluctuating light conditions (Bellasio and Griffiths 2014; Wang et al. 2014). Other anatomical and biochemical variations observed here might alter the hydraulic efficiency and growth rates of the different Andropogoneae (Osborne and Sack 2012). Overall, we conclude that, because of continuous adaptive reinforcement following a key physiological transition, descendants of a lineage sharing the derived trait should not all be considered as functionally equivalent.

## Conclusion

Using plastome and nuclear phylogenomics, we confirmed a rare Asian C$_{3}$ lineage, Jansenelleae, as sister to the C$_{4 }$Andropogoneae grasses. This opens new avenues for comparative analyses of C$_{4}$ evolution, which were explored here. The C$_{4}$ pathway in Andropogoneae most likely evolved in the Early Miocene between roughly 21 and 18 Ma, and many adaptive changes in C$_{4}$ enzymes happened during this 3-Myr period, while many more occurred during the next 18 million years of lineage diversification. The group including Andropogoneae apparently originated on the Indian subcontinent, and the evolutionary diversification of the C$_{4}$ phenotype after its origin might have facilitated the spread of Andropogoneae into novel niches and to different regions of the globe, contributing to the success of this emblematic group of savanna grasses.

## Supplementary Material

Data available from the Dryad Digital Repository: https://doi.org/10.5061/dryad.j6q573n7x.

## Funding

This work was supported by the Brazilian Research Council (CNPq) through a ”Science without Borders“ scholarship (grant number 201873/2014-1) and the European Research Council (grant ERC-2014-STG-638333) to M.E.B. J.H. and G.B. received support from the French excellence projects Labex CEBA (ANR-10-LABX-25-01) and Labex TULIP (ANR-10-LABX-0041). This work was performed within the framework of the PhyloAlps project, whose sequencing was funded by France Génomique (ANR-10-INBS-09-08). P.A.C. is funded by a Royal Society University Research Fellowship (URF120119). M.R.M. was supported by NSF grant DEB-11456884, M.R.D. by Dimensions NASA grant DEB-1342782, and DEB-1457748 to E.A.K. S.L. received funding from the LabexOSUG@2020 (ANR10 LABX56) and the project Origin-Alps (ANR-16-CE93-0004). Any opinions, findings, and conclusions or recommendations expressed in this material are those of the authors and do not necessarily reflect the views of the National Science Foundation.

## Appendix 1

Jansenelleae Voronts. tribus nov.

Type: *Jansenella* Bor, Kew Bull. 10: 96 (1955)

Included genera: *Chandrasekharania* V.J. Nair, V.S. Ramach. & Sreek.*, Jansenella* Bor

Description: Annuals with erect culms. Ligule shortly membranous. Leaf-blades lanceolate. Inflorescence shortly branched, appearing capitate. Spikelets laterally compressed, 2-flowered. Glumes 2, apically acuminate to shortly awned. Lower floret sterile, staminate, or bisexual. Lower lemma shortly awned, either entire (*Jansenella*) or awn arising between two erose apical lobes (*Chandrasekharania*). Lower palea present. Upper floret bisexual. Upper lemma awned from a bidentate apex, but variable in its shape and indumentum: either with two hair tufts, twisted dehiscent awn arising between long-acuminate lobes (*Jansenella*) or without hair tufts, short straight awn arising between two erose apical lobes (*Chandrasekharania*). Stamens 3. Grain ellipsoid; hilum punctiform.

Leaf anatomy: Outer and inner bundle sheaths present. More than four cells between consecutive veins. No distinctive cells. Starch storage in the chlorenchyma.

Distribution: India (including Assam and Sri Lanka), Myanmar and Thailand.
